# Mitochondrial Polymorphisms, in The D-Loop Area, Are Associated with Brain Tumors

**DOI:** 10.22074/cellj.2019.5947

**Published:** 2019-06-15

**Authors:** Donya Altafi, Soha Sadeghi, Hamed Hojatian, Maryam Torabi Afra, Safoura Pakizeh Kar, Mojtaba Gorji, Massoud Houshmand

**Affiliations:** 1 Molecular Biology Department, NourDanesh Institute of Higher Education, Esfahan, Iran; 2 Department of Biology, International University of Guilan, Guilan, Iran; 3 Department of Hematology and Oncology, Lorestan Medical University, Lorestan, Iran; 4 Department of Medical Genetics, National Institutes for Genetic Engineering and Biotechnology, Tehran, Iran; 5 Research Center, Knowledge University, Erbil, Kurdistan Region, Iraq

**Keywords:** Brain Tumor, D-Loop, Mitochondrial DNA

## Abstract

**Objective:**

This study was carried out to evaluate the relationship between mtDNA D-loop variations and the pathogenesis of a brain tumor.

**Materials and Methods:**

In this experimental study, 25 specimens of brain tumor tissue with their adjacent tissues from patients and 454 blood samples from different ethnic groups of the Iranian population, as the control group, were analysed by the polymerase chain reaction (PCR)-sequencing method.

**Results:**

Thirty-six variations of the D-loop area were observed in brain tumor tissues as well as the adjacent normal tissues. A significant difference of A750G (P=0.046), T15936C (P=0.013), C15884G (P=0.013), C16069T (P=0.049), T16126C (P=0.006), C16186T (P=0.022), T16189C (P=0.041), C16193T (P=0.045), C16223T (P=0.001), T16224C (P=0.013), C16234T (P=0.013), G16274A (P=0.009), T16311C (P=0.038), C16327T (P=0.045), C16355T (P=0.003), T16362C (P=0.006), G16384A (P=0.042), G16392A (P=0.013), G16394A (P=0.013), and G16477A (P=0.013) variants was found between the patients and the controls.

**Conclusion:**

The results indicated individuals with C16069T [odds ratio (OR): 2.048], T16126C (OR: 2.226), C16186T (OR: 3.586), G16274A (OR: 4.831), C16355T (OR: 7.322), and T16362C (OR: 6.682) variants with an OR more than one are probably associated with a brain tumor. However, given the multifactorial nature of cancer, more investigation needs to be done to confirm this association.

## Introduction

Brain tumors refer to all tumors in the central spinal canal or inside the cranium. All brain tumors are innately serious and fatal because of their infiltrative and invasive features in the confines of the intracranial cavity. According to the American Cancer Society, death estimation of Brain and other nervous system tumors is 6,150 (2%) per 100,000 in 2013. In 2013, 1310 cancer deaths amongst children between 0 to 14 years old were reported. Approximately, 25% of all cancers in children are due to brain and other central nervous system tumors (1-3) .

The human mitochondrial DNA (mtDNA) is a 16,569 base-pair-long double-strand DNA. Mitochondrial genome codes thirteen pivotal polypeptides of the respiratory chain enzyme complexes, which are located in the inner membrane of the mitochondria, along with two ribosomal RNAs (rRNAs) and twenty-two transfer RNAs (tRNAs) that are crucial for protein synthesis and intramitochondrial translation, respectively (4).

There are 10^3^ to 10^4^ copies of mtDNA per human cell (5). A 1.1 kb of noncoding displacement loop (D-loop) region is situated between the aforementioned genes. This region consists of heavy-strand and light-strand promoter regions which are essential for the mitochondrial transcription and replication process (4, 6, 7).

Due to insufficient DNA repair mechanisms, the absence of protective histone proteins, and a considerable level of the reactive oxygen species (ROS) production during oxidative phosphorylation (OXPHOS), mitochondrial DNA is susceptible to oxidative damage and carries significantly more mutations than nuclear DNA. In mtDNA, oxidative damages and the subsequent mutations can accumulate at a rate of 10-fold faster than nuclear DNA (8).

In the past, a myriad of somatic mtDNA mutations, including insertions, deletions, point mutations, and altered mtDNA copy numbers, have been detected in numerous human cancers (9). In the mitochondrial genome, some of these mutations lead to functional alterations in its encoded proteins or missense mRNA transcription (10). In the coding region, the common deletion region (CD) (4,977 bp deletion) is the most frequent (11). It has been shown that large deletions in the mitochondrial genome are accumulated in subjects with heteroplasmic mtDNA mutations and healthy elders (12).

The somatic variations in the D-loop area may be associated with the diminution of mtDNA copy numbers as well (13). The D-loop area of Mitochondrial DNA is a polymorphic region (14). The most of the mtDNA mutations occurring in D-loop are linked to human malignancies (13). Therefore, studying mtDNA mutations in various tumor cells is pivotal for understanding the relationship between mtDNA D-loop mutations and the initiation and progression of a tumor (15).

## Materials and Methods

### Subjects

In this experimental study, 25 samples of brain tumors and adjacent tissues (2 women and 23 men in the age range of 28 to 70 years old) were collected (Table 1). Furthermore, 454 blood samples from healthy controls were included; these samples are from 100 random individuals, belonging to17 ethnicities of the Iranian population. All of the samples were collected from the Tehran Special Medical Center. Tumor tissues and adjacent ones were quickly frozen by liquid nitrogen and transferred to a -80˚C freezer. The cancer diagnosis was confirmed via histological analysis. In the control group, the exclusion criterion was metabolic diseases, history of cancer, and any mitochondrial DNA related diseases which might affect the mtDNA.

**Table 1:** Age and histological features of brain tumors in patients


Sample	Age	Clinical data	Diagnosis	Gender

1	53	Left frontal brain tumor	Astrocytoma	Male
2	53	Right fronto-temporal brain tumor	Giant Cell Glioblastoma	Male
3	40	A right parieto-occipital brain tumor	Oligodendroglioma	Male
4	48	Right temporal brain tumor	Astrocytoma	Male
5	58	Left frontotemporal brain tumor	Astrocytoma	Male
6	33	Intraventicular brain tumor	Ependymoma	Male
7	56	Right temporal brain tumor	Astrocytoma	Male
8	51	Right temporal brain tumor	Glioblastoma multiform	Male
9	55	Left temporal brain tumor	Astrocytoma	Male
10	57	Right frontoparietal brain tumor	Glioblastoma multiform	Male
11	65	Left temporal brain tumor	Astrocytoma	Male
12	42	Right parieto-occipital brain tumor	Oligodendroglioma	Male
13	70	Right frontoparietal brain tumor	Glioblastoma multiform	Female
14	54	Right frontal brain tumor	Glioblastoma multiform	Male
15	58	A cerebral tumor	Glioblastoma multiform	Male
16	53	Right temporal brain tumor	Glioblastoma multiform	Male
17	63	Right temporal brain tumor	Glioblastoma multiform	Male
18	40	A cerebral tumor	Glioblastoma multiform	Male
19	28	Left frontal brain tumor	Glioblastoma multiform	Male
20	55	A right frontoparietal brain tumor	Oligodendroglioma	Male
21	63	Right frontal brain tumor	Glioblastoma multiform	Male
22	32	Left frontal cerebral tumor	Glioblastoma multiform	Male
23	32	Cerebral tumor	Astrocytoma	Male
24	56	Right temporal brain tumor	Astrocytoma	Female
25	36	Left temporal brain tumor	Astrocytoma	Male


### DNA extraction

Genomic DNA of the tumor and the adjacent tissues were isolated by DNA extraction kit (Qiagen, Netherlands) and blood samples of the controls. which were collected in Ethylenediaminetetraacetic acid (EDTA)-containing tubes, were extracted using Diatom DNA extraction kit (Gene Fanavaran, Iran) and their quantity and quality were analyzed by NanoDrop 2000 UV-Vis Spectrophotometer (Thermo Fisher Scientific) and agarose gel electrophoresis, respectively.

### Mitochondrial D-loop genotyping

Primers for amplifying of the mtDNA D-loop region were adapted from Seyedhassani et al. (16) and Shakhssalim et al. (17).

The volume of a PCR reaction was 25 μl and contained 50- 100 ng of the DNA, 0.8 μl of the primers, 0.8 μl of MgCl2, 0.5 μl of dNTPs 10 mM, 2.5 μl of PCR buffer (10X), and 0.3 μl of Taq DNA polymerase (Roche Applied Sciences, Germany). Moreover, the PCR procedure was carried out according to the following protocol: pre-denaturation phase (5 minutes at 94˚C), followed by 35 cycles of the shorter denaturation phase (50 seconds at 94˚C), annealing step (50 seconds at 55˚C), the extension step (50 seconds at 72˚C), The final stage is the extension phase for 10 minutes at 72˚C. Each amplified fragment subsequently was sequenced using an ABI PRISM 3730 sequence analyzer (Macrogen, Korea). The acquired sequencing data were evaluated using Finch TV.

### Statistical analysis

Finch TV version 1.4.0 was used to edit and align the sequences. The NCBI blast was used as a reference. The statistical analysis was carried out using IBM SPSS statistics for windows, version 24.0 (IBM Corp., Armonk, NY, USA). The chi-square was used to evaluate the deviation from the Hardy-Weinberg equilibrium. This association between a brain tumor and D-loop variants was evaluated using SPSS version 22. A P<0.05 was considered statistically significant

## Results

In the present study, tissue samples from 25 subjects with brain tumors and 454 blood samples from the control group were genotyped for D-loop region variants. The subsequent results showed 36 variations in the D-loop area (Table 2), most of which were already reported under MITOMAP. One hundred and fifteen variations of the D-loop region were found in the control groups (Table 3). However, 34 mutations in the patients and 103 mutations in the control were reported in advance, and 2 mutations in the patients and 12 mutations in the control were novel mutations (Table 4). All variations were single nucleotide substitutions and the majority of them were C→T. (33.33%) T→C (30.55%) G→A (22.22%) A→C (2.77%) C→G (2.77%). The D-loop region variant distributions in the controls and the patients are shown in Table S1 (See Supplementary Online Information at www. celljournal.org). The significant associations between variants and brain tumor risk (P<0.05) are shown in Table 5.

**Table 2:** List of D-loop region variants in the patient group (tissue sample)



A152G	T195C	T204C	G207A	A263G	T489C	A750G	C15884G	G15928A	T15936C^*^
C16069T	T16126C	G16145A	C16148T	T16172C	C16186T	T16189C	C16193T	C16223T	T16224C
C16234T	C16256T	C16261T	C16270T	G16274A	C16292T	T16311C	A16318C	C16327T	C16355T
T16362C	G16384A	G16392A	G16394A	G16477A	T16519C				


*; Shows unreported mutations near the D-loop region.

**Table 3:** List of D-loop region variants in control group (blood sample)



A152G	T195C	T204C	G207A	A263G	T489C	A750G	A15908G	T15924C	C15927T
C15928T	G15946A	G16004C^*^	A16017G	A16069G	G16071A	A16075G	C16084T	A16086G	A16087G^*^
A16093G	G16095A	G16114A	G16124A^*^	A16126G	A16128G^*^	C16129T	G16131A^*^	C16145T	G16148A
C16149T	G16150A	T16162C	T16163C	A16172G	G16174A	G16176A	A16177G	A16179G	G16184A
G16186A	C16187T	A16189G	G16192A	G16193A	G16197A	G16201A	T16203C	A16209G	A16217G
A16223G	G16233A	G16236A	G16242A	A16243G	G16245A	A16248G	A16249G	G16256A	G16257A
G16259A	G16261A	G16262A	T16265C	C16266T	G16270A	A16271G	A16272G	C16274T	G16278A
A16286G	G16287A	A16289G	G16290A	G16291A	G16292A	T16293G	G16294A	G16295A	G16296A
A16299G	A16304G	T16309C	A16311G	G16315A^*^	T16316C	T16318A	C16319T	A16323G	A16325G
A16334G	C16339T	A16342G	T16343C	A16344G^*^	A16352G	G16354T	G16355A	A16356G	A16359G
G16360A	A16362G	G16364A	C16390T	C16391T	C16398T	T16399C	A16468G	G16478C	G16481A^*^
G16488A	T16497C	T16519C	C16526T	G16527A					


*; Shows unreported variations in D-loop region and adjacent sequences.

**Table 4:** List of unreported variants in patient and control groups



A15499G	G16004C	A16087G	G16124A	A16128G	G16131A	A16179G	G16233A	A16286G	G16315A
A16344G	G16481A								


**Table 5:** The variations statistical analysis and associations between them whit brain tumour risk


Odds ratio	Chi-square tests	P value	Control (%)	Patients (%)	Variant

0.94	6.186^a^	0.013	0	6.25	T15936C
0.94	6.186^a^	0.013	0	6.25	C15884G
1	0.000^a^	1	6.38	6.25	G15928A
2.048	3.854^a^	0.049	13.87	25	C16069T
2.226	7.490^a^	0.006	30.83	50	T16126C
1.57	1.339^a^	0.247	12.99	18.75	G16145A
1.532	0.421^a^	0.516	3.96	6.25	C16148T
1.532	0.421^a^	0.516	4.18	6.25	T16172C
3.586	5.207^a^	0.022	3.52	12.5	C16186T
0.457	4.190^a^	0.041	22.9	12.5	T16189C
6.319	3.701^a^	0.045	1.32	6.25	C16193T
0.318	10.351^a^	0.001	31.71	12.5	C16223T
0.94	6.186^a^	0.013	0.22	6.25	T16224C
0.94	6.186^a^	0.013	0.88	6.25	C16234T
1.532	0.421^a^	0.516	4.4	6.25	C16256T
1.898	2.510^a^	0.113	10.57	18.75	C16261T
2.064	1.047^a^	0.306	2.6	6.25	C16270T
4.831	6.793^a^	0.009	2.86	12.5	G16274A
2.064	1.047^a^	0.306	2.64	6.25	C16292T
0.362	4.310^a^	0.038	14.75	6.25	T16311C
1.213	0.96^a^	0.756	4.62	6.25	A16318C
6.319	3.701^a^	0.045	0.66	6.25	C16327T
7.322	8.721^a^	0.003	2.2	12.5	C16355T
6.682	7.680^a^	0.006	9.25	18.75	T16362C
2.372	4.153^a^	0.042	0	6.25	G16384A
0.94	6.186^a^	0.013	0.22	6.25	G16392A
0.94	6.186^a^	0.013	0	6.25	G16394A
0.94	6.186^a^	0.013	0	6.25	G16477A
0.747	1.017^a^	0.313	62.55	56.25	T16519C
1.047	0.023^a^	0.87	30.8	32	A152G
0.5	2.94^a^	0.08	21.4	12	T195C
1.37	0.47^a^	0.48	9.3	12	T204C
1.81	1.45^a^	0.22	7	12	G207A
0	0	0	100	100	A263G
1.34	0.741^a^	0.38	19.1	24	T489C
0.39	3.979^a^	0.046	93.3	84	A750G


^a^; 0 blocks (0.0%) have considered rate less than 5. The minimum envisaged rate is 31.50.

## Discussion

Studies have shown that the accumulation of mitochondrial DNA variations and mitochondrial instability contributes to several diseases, including cancer. The mitochondrial D-loop area has pivotal functions in transcription, replication, and mtDNA organization. Presence of two critical factors makes mitochondrial genome prone to different mutations: ROS and low-level of DNA repair system in mitochondria (18). However, the D-loop region is even more susceptible to the mutations than the rest of the human mitochondrial genome (12).

It has been demonstrated that the expansion of poly-C repeats in highly variable regions (HVR), adjacent to the D-loop and tRNA phenylalanine, increases the copy number; homoplasmy and loss of mitochondrial heteroplasmy act like cancer suppressor genes and will cause brain cancer and other cancers in humans (19).

Recent studies demonstrate that mitochondrial D-loop mutations play an important role in Huntington’s disease (20), as well as various cancers, including brain tumors. Consequently, in this survey was hypothesised that particular mutations in the D-loop region might be related with brain tumor risk.

Twenty-five tissue samples of brain tumors were assessed. As it is not possible to use brain tissues of healthy individuals, we used 454 blood samples as the control. A significant difference between the cancer tissue and the healthy tissue was observed. Also, we studied the mutations reported in MITOMAP in more detail.

It has been proven that D-loop variants are associated with different disease: T195C variant with glaucoma (21) and G15884C variant with pancreatic cancer (22) and G15928A with MS, recurrent idiopathic abortion and AD (23) and C16069T with bladder cancer (24) and T16126C with Huntington’s disease (20) and C16172T with head and neck cancer (25) and T16189C with prostate cancer (26) and C16193T with ovarian cancer (27) and C16223T with cancer, and Huntington’s (20) and G16274A linked to prostate cancer (28) and C16292T related with breast and ovarian cancer (27) and T16311C with prostate cancer (28) and T16519C with glioblastoma, migraines (29). Although T204C (30), G207A (28), A263G (31), T489C (25), A750G (21), G16145A (16), C16148T (32), C16186T (33), T16224C, C16234T, C16256T (34), C16270T, C16261T (35, 36), C16327T (37), C16355T (38), T16362C (39), G16384A (16), G16392A, G16394A, G16477A (40), were associated with various cancers and were reported in MITOMAP but they were not associated with the disease.

In this experimental study, A750G, T15936C, C15884G, C16069T, T16126C, C16186T, T16189C, C16193T, C16223T, T16224C, C16234T, G16274A, T16311C, C16327T, C16355T, T16362C, G16384A, G16392A, G16394A, G16477A variants had statistically significant different frequencies between patient and control groups. Thus, it seems that they are associated with brain tumors. Among the mentioned polymorphisms, C16069T, T16126C, C16186T, G16274A, C16355T, and T16362C had a greater odd ratio (OR) than the rest. C16355T polymorphism had the most significant association with the disease in risk.

It should be noted that A152G, T204C, G207A, A263G, T489C, A750G, G15928A, G16145A, C16148T, T16172C, C16256T, C16261T, C16270T, C16292T, A16318C, and T16519C polymorphisms were not associated with the disease risk. Although, they have significant roles in other disease pathogenesis as they have been reported on MIT OMAP.

In Lorr ethnicity, T15936C, C15884G, G15928A, T16126C, C16148T, T16172C, C16186T, C16186T, C16193T, C16223T, T16224C, C16234T, C16256T, C16292T, T16311C, T16362C, G16384A, G16392A, G16394A, and G16477A polymorphisms were associated with brain tumors. T16126C and T16362C polymorphisms had a higher odds ratio than the rest which indicates they are more associated with this disease.

C16069T, G16145A, T16189C, C16261T, C16270T, A16318C, C16327T, and T16519C polymorphisms were not associated with the disease in Lorr ethnicity. However, the mentioned polymorphisms are associated with other disease pathogenesis as they reported on MITOMAP.

In Fars ethnicity, T15936C, C15884G , C16069T, T16126C, C16193T, T16224C, C16234T, C16261T, C16292T, C16327T, C16355T, G16384A, G16392A,G16394A, and G16477A variants were associated with the disease, C16069T, T16126C, C16186T, G16274A, C16355T, and T16362C polymorphisms had greater odds ratio than the rest, and G15928A, G16145A, C16148T, T16172C, C16186T, T16189C, C16223T, C16256T, C16270T, G16274A, T16311C, A16318C, T16362C, and T16519C variants were not associated with the disease.

## Conclusion

Base on the odds ratio result, C16069T, T16126C, C16186T, G16274A, C16355T, and T16362C alterations were significantly associated with brain tumor. Among these variants, C16355T with odds ratio 7.322 was the strongest one.

## Supplementary PDF


