# Supplementary material for: Mitochondrial Polymorphisms, in The D-Loop Area, Are Associated with Brain Tumors
Source: Cell J. 2019 Jun 15;21(3):350–6. doi: 10.22074/cellj.2019.5947 (PMC6582428; doi:10.22074/cellj.2019.5947)
Supplement: Supplementary file 1 [file Cell-J-21-350-s01.pdf]

CELL JOURNAL (Yakhteh)

www.celljournal.org

Supplementary Information for

Mitochondrial Polymorphisms, in The D-Loop Area, Are Associated with Brain Tumors

Donya Altafi, M.Sc.[1]*, Soha Sadeghi. M.Sc.[1], Hamed Hojatian, M.Sc.[1], Maryam Torabi Afra, M.Sc.[1], Safoura Pakizeh Kar, M.Sc.[2], Mojtaba Gorji, Ph.D.[3], Massoud Houshmand, Ph.D.[4, 5]*

1. Molecular Biology Department, NourDanesh Institute of Higher Education, Esfahan, Iran
2. Department of Biology, International University of Guilan, Guilan, Iran
3. Department of Hematology and Oncology, Lorestan Medical University, Lorestan, Iran
4. Department of Medical Genetics, National Institutes for Genetic Engineering and Biotechnology, Tehran, Iran
5. Research Center, Knowledge University, Erbil, Kurdistan Region, Iraq

**Corresponding Addresses: P.O.Box: 1483614681, Molecular Biology Department, NourDanesh Institute of Higher Education, Esfahan, Iran*
P.O.Box: 14965/161, Department of Medical Genetics, National Institutes for Genetic Engineering and Biotechnology, Tehran, Iran
Emails: donya.altafi@gmail.com, massoudh@nigeb.ac.ir

Table S1: D-loop region variant distributions in controls and cases

Ethnicity	Number	A152G	T195C	T204C	G207A	A263G	T489C	A750G
Arab		21.70%	17.30%	4.30%	8.70%	100%	17.30%	95.60%
	23	5	4	1	2	23	4	22
Armenian		22.20%	11.10%	11.10%	11.10%	100%	11.10%	94.40%
	18	4	2	2	2	18	2	17
Asurian		0%	0%	0%	0%	0%	0%	0%
	16	0	0	0	0	0	0	0
Azari		63.60%	22.70%	13.60%	9.10%	100%	13.60%	95.40%
	24	14	5	3	2	22	3	21
Baluch		0%	0%	0%	0%	0%	0%	0%
	12	0	0	0	0	0	0	0
Bandari		0%	0%	0%	0%	0%	0%	0%
	30	0	0	0	0	0	0	0
Esfahan		12.50%	18.75%	18.75%	0%	100%	12.50%	87.50%
	15	2	3	3	0	16	2	14
Kerman		0%	0%	0%	0%	0%	0%	0%
	25	0	0	0	0	0	0	0
Mashhad		39.10%	30.40%	8.70%	13%	100%	30.40%	100%
	24	9	7	2	3	23	7	23
Shiraz		30.40%	13%	17.40%	13%	100%	21.70%	95.60%
	22	7	3	4	3	23	5	22
Yazd		29.10%	37.50%	8.30%	4.10%	100%	12.50%	83.30%
	24	7	9	2	1	24	3	20
Gilani		29.10%	37.50%	8.30%	4.10%	100%	16.60%	91.60%
	24	7	9	2	1	24	4	22
Jews		35.10%	10.80%	0%	2.70%	100%	18.90%	100%
	38	13	4	0	1	37	7	37
Kurd		29.10%	20.80%	8.30%	4.10%	100%	20.80%	87.50%
	25	7	5	2	1	24	5	21
Lur		18.10%	18.10%	13.60%	9.10%	100%	31.80%	90.90%
	23	4	4	3	2	22	7	20
Mazani		0%	0%	0%	0%	0%	0%	0%
	23	0	0	0	0	0	0	0
Mixed Tehran		0%	0%	0%	0%	0%	0%	0%
	88	0	0	0	0	0	0	0
Total(Controls)		30.80%	21.40%	9.30%	7%	100%	19.10%	93.30%
	454	79	55	24	18	256	49	239
patients		32%	12%	12%	12%	100%	24%	84%
	25	8	3	3	3	25	6	21

Table S1: Continued

Ethnicity	Number	T15936C	C15884G	G15928A	C16069T	T16126C	G16145A	C16148T
Arab		0%	0%	0%	13.04%	34.78%	17.39%	8.69%
	23	0	0	0	3	8	4	2
Armenian		0%	0%	0%	16.66%	16.66%	22.22%	0%
	18	0	0	0	3	3	4	0
Asurian		0%	0%	0%	25%	31.25%	6.25%	0%
	16	0	0	0	4	5	1	0
Azari		0%	0%	12.50%	12.50%	25%	0%	4.16%
	24	0	0	3	3	6	0	1
Baluch		0%	0%	0%	16.66%	16.66%	8.33%	0%
	12	0	0	0	2	2	1	0
Bandari		0%	0%	0%	13.33%	23.33%	10%	3.33%
	30	0	0	0	4	7	3	1
Esfahan		0%	0%	6.66%	6.66%	20%	13.33%	0%
	15	0	0	1	1	3	2	0
Kerman		0%	0%	8%	0%	16%	4%	0%
	25	0	0	2	0	4	1	0
Mashhad		0%	0%	16.66%	16.66%	54.16%	12.50%	4.16%
	24	0	0	4	4	13	3	1
Shiraz		0%	0%	18.18%	9.09%	40.90%	13.63%	0%
	22	0	0	4	2	9	3	0
Yazd		0%	0%	0%	12.50%	33.33%	16.66%	12.50%
	24	0	0	0	3	8	4	3
Gilani		0%	0%	0%	16.66%	25%	16.66%	8.33%
	24	0	0	0	4	6	4	2
Jews		0%	0%	0%	13.15%	21.05%	10.52%	7.89%
	38	0	0	0	5	8	4	3
Kurd		0%	0%	12%	32%	40%	20%	8%
	25	0	0	3	8	10	5	2
Lur		0%	0%	0%	21.73%	34.78%	26.08%	0%
	23	0	0	0	5	8	6	0
Mazani		0%	0%	21.73%	17.39%	43.47%	17.39%	0%
	23	0	0	5	4	10	4	0
Mixed Tehran		0%	0%	7.95%	9.09%	34.09%	11.36%	3.40%
	88	0	0	7	8	30	10	3
Total(Controls)		0%	0%	6.38%	13.87%	30.83%	12.99%	3.96%
	454	0	0	29	63	140	59	18
patients		8%	8%	8%	24%	52%	20%	8%
	25	2	2	2	6	13	5	2

Table S1: Continued

Ethnicity	Number	T16172C	C16186T	T16189C	C16193T	C16223T	T16224C	C16234T
Arab		4.34%	0%	30.43%	0%	30.43%	0%	0%
	23	1	0	7	0	7	0	0
Armenian		11.11%	0%	22.22%	5.55%	27.77%	0%	0%
	18	2	0	4	1	5	0	0
Asurian		0%	0%	31.25%	0%	6.25%	0%	0%
	16	0	0	5	0	1	0	0
Azari		0%	4.16%	16.66%	4.16%	25%	0%	0%
	24	0	1	4	1	6	0	0
Baluch		0%	8.33%	16.66%	0%	8.33%	0%	0%
	12	0	1	2	0	1	0	0
Bandari		3.33%	10%	40%	0%	43.33%	0%	0%
	30	1	3	12	0	13	0	0
Esfahan		0%	0%	46.66%	0%	46.66%	0%	0%
	15	0	0	7	0	7	0	0
Kerman		4%	0%	32%	4%	36%	0%	4%
	25	1	0	8	1	9	0	1
Mashhad		12.50%	8.33%	25%	0%	37.50%	0%	0%
	24	3	2	6	0	9	0	0
Shiraz		4.54%	4.54%	22.72%	4.54%	45.45%	0%	0%
	22	1	1	5	1	10	0	0
Yazd		8.33%	8.33%	25%	4.16%	33.33%	0%	4.16%
	24	2	2	6	1	8	0	1
Guilani		4.16%	0%	8.33%	0%	37.50%	0%	8.33%
	24	1	0	2	0	9	0	2
Jews		0%	0%	15.78%	0%	23.68%	0%	0%
	38	0	0	6	0	9	0	0
Kurd		4%	4%	24%	0%	28%	4%	0%
	25	1	1	6	0	7	1	0
Lur		0%	0%	4.34%	0%	26.08%	0%	0%
	23	0	0	1	0	6	0	0
Mazani		8.69%	0%	17.39%	0%	34.78%	0%	0%
	23	2	0	4	0	8	0	0
Mixed Tehran		4.54%	5.68%	21.59%	1.13%	32.95%	0%	0%
	88	4	5	19	1	29	0	0
(Total(Controls		4.18%	3.52%	22.90%	1.32%	31.71%	0.22%	0.88%
	454	19	16	104	6	144	1	4
Patients		8%	12%	12%	8%	12%	8%	8%
	25	2	3	3	2	3	2	2

Table S1: Continued

Ethnicity	Number	C16256T	C16261T	C16270T	G16274A	C16292T	T16311C	A16318C
Arab		4.34%	13.04%	0%	8.69%	0%	13.04%	8.69%
	23	1	3	0	2	0	3	2
Armenian		11.11%	11.11%	5.55%	11.11%	0%	16.66%	0%
	18	2	2	1	2	0	3	0
Asurian		0%	6.25%	0%	0%	0%	0%	0%
	16	0	1	0	0	0	0	0
Azari		0%	0%	0%	0%	4.16%	20.83%	0%
	24	0	0	0	0	1	5	0
Baluch		0%	8.33%	0%	8.33%	0%	0%	0%
	12	0	1	0	1	0	0	0
Bandari		3.33%	10%	0%	0%	6.66%	16.66%	0%
	30	1	3	0	0	2	5	0
Esfahan		0%	26.66%	13.33%	0%	0%	33.33%	0%
	15	0	4	2	0	0	5	0
Kerman		4%	4%	4%	4%	0%	4%	4%
	25	1	1	1	1	0	1	1
Mashhad		0%	8.33%	0%	0%	8.33%	12.50%	8.33%
	24	0	2	0	0	2	3	2
Shiraz		13.63%	9.09%	4.54%	13.63%	4.54%	9.09%	4.54%
	22	3	2	1	3	1	2	1
Yazd		0%	8.33%	0%	0%	4.16%	12.50%	4.16%
	24	0	2	0	0	1	3	1
Guilani		4.16%	12.50%	4.16%	4.16%	4.16%	8.33%	4.16%
	24	1	3	1	1	1	2	1
Jews		5.26%	10.52%	2.63%	0%	2.63%	15.78%	10.52%
	38	2	4	1	0	1	6	4
Kurd		4%	20%	4%	0%	8%	24%	4%
	25	1	5	1	0	2	6	1
Lur		17.39%	21.73%	4.34%	0%	0%	39.13%	8.69%
	23	4	5	1	0	0	9	2
Mazani		0%	17.39%	0%	0%	4.34%	8.69%	4.34%
	23	0	4	0	0	1	2	1
Mixed Tehran		4.54%	6.81%	3.40%	3.40%	0%	13.63%	5.68%
	88	4	6	3	3	0	12	5
(Total(Controls		4.40%	10.57%	2.60%	2.86%	2.64%	14.75%	4.62%
	454	20	48	12	13	12	67	21
patients		8%	20%	8%	12%	8%	8%	8%
	25	2	5	2	3	2	2	2

Table S1: Continued

Ethnicity	Number	C16327T	C16355T	T16362C	G16384A	C16392T	C16394T	G16477A	T16519C
Arab		0%	8.69%	17.39%	0%	0%	0%	0%	65.21%
	23	0	2	4	0	0	0	0	15
Armenian		0%	11.11%	0%	0%	0%	0%	0%	55.55%
	18	0	2	0	0	0	0	0	1000%
Asurian		0%	0%	6.25%	0%	0%	0%	0%	62.50%
	16	0	0	1	0	0	0	0	10
Azari		0%	4.16%	8.33%	0%	0%	0%	0%	79.16%
	24	0	1	2	0	0	0	0	19
Baluch		0%	0%	0%	0%	0%	0%	0%	75%
	12	0	0	0	0	0	0	0	9
Bandari		0%	0%	6.66%	0%	0%	0%	0%	53.33%
	30	0	0	2	0	0	0	0	16
Esfahan		0%	0%	20%	0%	0%	0%	0%	46.66%
	15	0	0	3	0	0	0	0	7
Kerman		0%	0%	12%	0%	0%	4%	0%	0%
	25	0	0	3	0	0	1	0	0
Mashhad		0%	0%	4.16%	0%	0%	0%	0%	75%
	24	0	0	1	0	0	0	0	18
Shiraz		0%	0%	22.72%	0%	4.54%	9.09%	0%	68.18%
	22	0	0	5	0	1	2	0	15
Yazd		0%	8.33%	4.16%	0%	0%	4.16%	0%	70.83%
	24	0	2	1	0	0	1	0	17
Guilani		4.16%	0%	4.16%	0%	0%	0%	0%	66.66%
	24	1	0	1	0	0	0	0	16
Jews		0%	2.63%	5.26%	0%	0%	0%	0%	71.05%
	38	0	1	2	0	0	0	0	27
Kurd		4%	4%	8%	0%	0%	0%	0%	72%
	25	1	1	2	0	0	0	0	18
Lur		4.34%	0%	8.69%	0%	0%	0%	0%	65.21%
	23	1	0	2	0	0	0	0	15
Mazani		0%	0%	0%	0%	0%	0%	0%	56.52%
	23	0	0	0	0	0	0	0	13
Mixed Tehran		0%	1.13%	14.77%	0%	0%	0%	0%	67.04%
	88	0	1	13	0	0	0	0	59
(Total(Controls		0.66%	2.20%	9.25%	0%	0.22%	0%	0%	62.55%
	454	3	10	42	0	1	4	0	284
patients		8%	12%	20%	8%	8%	8%	8%	56%
	25	2	3	5	2	2	2	2	14
